# The mediating effect of cognitive and emotional processing on PTSD and depression symptoms reduction in women victims of IPV

**DOI:** 10.3389/fpsyg.2022.1071477

**Published:** 2022-12-23

**Authors:** Rossella Procaccia, Marco Castiglioni

**Affiliations:** ^1^Faculty of Psychology, eCampus University, Novedrate, Italy; ^2^Department of Human Sciences “R. Massa,” University of Milano-Bicocca, Milan, Italy

**Keywords:** depression, PTSD, expressive writing, intimate partner violence, cognitive processing, emotional processing, making sense

## Abstract

Intimate partner violence (IPV) is a serious social, physical and mental health issue. Women victims of IPV can develop short- and long-term consequences such as depression and post-traumatic stress disorder (PTSD). Where trauma has been incurred, standard psychotherapies may usefully be complemented by interventions based on expressive writing (EW). Numerous studies have explored the mechanisms underpinning improvement after writing, focusing on the cognitive and emotional processing of traumatic experiences. The aims of this study were to evaluate changes in PTSD and depression symptoms following EW and to examine the mediating effect of emotional and cognitive processing on symptom reduction in subjects who engaged in EW. Seventy-seven abused women (mean age = 41.43, SD = 10.75) were randomly assigned to a three-session expressive writing condition (*n* = 43) or a neutral writing condition (*n* = 34). Psychological distress (PTSD and depression) was assessed both before and after the writing sessions. Linguistic inquiry word count software was used to analyze the women’s narratives in relation to emotional processing (positive and negative emotions) and cognitive processing (insight and causal attributions). The mediation model indicated that the reduction in depression was fully mediated by negative emotion processing and partially mediated by cognitive processing, while the reduction in PTSD was partially mediated by negative emotion processing and fully mediated by cognitive processing. No effect of positive emotion processing was found. The clinical implications are discussed.

## Introduction

Intimate partner violence (IPV) is a serious public health problem that affects millions of women, and is associated with severe physical and psychological health outcomes (Koopman et al., 2005). The World Health Organization (2010, p. 11) defines IPV as “*behaviour within an intimate relationship that causes physical, sexual or psychological harm, including acts of physical aggression, sexual coercion, psychological abuse and controlling behaviours*”. IPV may be inflicted by current or former partners, who are male in 90% of cases (Holmes et al., 2007). Official data (ISTAT, 2015) suggests that in Italy about 2,800,000 women (aged 16–70 years) have suffered one or more episodes of sexual or physical violence at the hands of a partner or cohabitant (Troisi, 2018). IPV victims report physical and mental health problems such as chronic pain, sleep disorders, poor overall health, depression, PTSD, anxiety, substance abuse, suicidality and self-harm (Dillon et al., 2013; Mazza et al., 2021). Given the incidence and severity of IPV-related consequences, clinicians and researchers seek to provide victims of IPV with valid psychological interventions. However, the victims are not quick to seek psychological support, likely because they primarily need safety intervention. Where trauma has been incurred, standard psychotherapies may usefully be complemented by expressive writing (EW) interventions (Pennebaker, 1997). Multiple authors have documented the benefits of writing about stressful and traumatic experiences over multiple sessions of up to 20 min. EW is associated with reduced physical pain, doctor visits, health problems, depression and distress (Pennebaker and Francis, 1996; Smyth et al., 1999). It has been applied with survivors of traumatic events including childhood sexual abuse (Foà et al., 1995; Meston et al., 2013), rape (Kearns et al., 2010) and the Holocaust (Pennebaker et al., 1989), and more recently with the general population (Negri et al., 2020), women undergoing assisted reproductive treatment (Renzi et al., 2019, 2020) and frontline healthcare workers during the COVID-19 emergency (Procaccia et al., 2021). EW studies have also been conducted with IPV survivors, but with conflicting outcomes to date: some found that depression and PTSD improved after writing (Koopman et al., 2005), while other follow-up studies found that women in an EW group were more distressed after 5 weeks than were control subjects (Gidron et al., 1996; Smyth et al., 2001). Some research suggests that subjects whose psychological distress is more severe prior to engaging in EW benefit more from it. Still other studies found that outcomes did not vary as a function of baseline values (Koopman et al., 1999). Hence, more systematic empirical research is required to establish whether writing can help to resolve trauma, in whom, and *via* what mechanisms.

Numerous studies have used linguistic inquiry word count (LIWC; Pennebaker and Francis, 1996) to explore the relationship between health improvements and linguistic markers of cognitive and emotional processing in trauma narratives. Greater cognitive shifts in writing are correlated with bigger health improvements (Warner et al., 2005). Cognitive changes from the first to the last writing sessions are inferred from increased usage of words such as “*why*” “*reason*” “*realize*” and “*understand*,” which reflect more “causal” and “insightful” thinking (Pennebaker, 2000). Thus, changing narrative structures flag deeper reflection about the meanings and causal nature of events, demonstrating that repeated narrative construction fosters cognitive processing. Some authors have also examined the role of emotional processing in trauma narratives (Pennebaker and Francis, 1996). Increased expression of positive emotion (reflected in the use of words such as “*happiness*” and “*joy*”) and moderate use of negative emotional lexicon (“*sad*,” “*guilt*” and “*angry*”) are linked with greater improvements in physical health (Pennebaker et al., 1997). However, few studies have examined the narratives of IPV survivors (Southern, 2013). Thus, the aims of this study were to: (a) evaluate whether expressive writing helps mitigate PTSD and depression in female victims of IPV; (b) explore the mediating effect of cognitive and emotional processing on changes in symptomatology. We hypothesized that women who took part in EW sessions would display a greater reduction in symptoms than a control group who completed a neutral writing task (H1); and that cognitive and emotional processing would mediate the effect of baseline values on reductions in symptoms (H2).

## Materials and methods

### Participants

Participants were recruited through services for abused women settled in Northern and Central Italy. Inclusion criteria included: having been a victim of IPV; being over 18 years of age; displaying adequate proficiency in written and spoken Italian; currently living in safe conditions (separated from the abusive partner for at least 30 days; not cohabiting with the partner for at least 6 months). Seventy-eight women joined the study. Their mean age was 41.43 years (SD = 10.75); the majority were Italian, with a medium-high level of education and in employment. Most had been married or stabling cohabiting and had children; they were victims of chronic and multiple forms of abuse. Participants displayed strong PTSD symptoms as measured using the LASC cut-off values (see King et al., 1995, p. 14); half were moderately or severely depressed (see Table 1). Data were collected between January and August 2022.

**Table 1:** Demographics.

Total number	77	
Age (years)		
Mean (SD)	41.43 (10.75)	
Min–max	19	59
Nationality		
Italian	56	72.7%
Not Italian	21	27.3%
Education		
Middle school license	35	45.5%
Degree	20	26.0%
Post-graduate degree	22	28.5%
Occupational status		
Employed	58	75.3%
Unemployed	19	24.7%
Marital status		
Married or cohabiting	61	79.22%
Stable partner	16	20.78%
Children		
Children	59	76.6%
No children	18	23.4%
Year of victimization		
<1 year	11	14.28%
>1 year	66	85.72%
Type of victimization		
Psychological abuse	77	100%
Physical abuse	63	81.80%
Sexual abuse	56	72.72%
More than one type of abuse	70	90.90%
PTSD		
Mean (SD)	28.14 (14.52)	
Min–max	0	60.00
Depression		
Minimal range	20	26.0%
Mild depression	17	22.1%
Moderate depression	15	19.5%
Severe depression	25	32.5%

### Procedure

In the pre-writing phase, participants received an envelope containing a briefing about the study, consent forms, a socio-demographic questionnaire, and all the other research questionnaires (Time 1). The briefing warned of the risks associated with the study, including distress from recalling traumatic experiences. The participants were aware that they could withdraw at any time. They completed the questionnaires individually at home and then received another envelope containing instructions for the writing task. Following the standard narrative research protocol developed by Pennebaker and Francis (1996), the women were randomly assigned to either the “expressive writing” group, and told to write about their traumatic experiences with a focus on exploring their deepest emotions and feelings about them (EW, *n* = 44); or to a “neutral writing” group, with instructions to write about their traumatic experiences but focusing only on facts and events (NW, *n* = 34). Beginning 3 days later, the participants were invited to write for up to 20 min per day on three consecutive days in their own homes. One week later, they were invited to complete the study questionnaires for the second time (Time 2). The study complied with the Ethics Code of Italian Psychologists and was approved by the Ethics Committee of eCampus University. All participants provided written informed consent. Participants’ personal information was handled in compliance with the General Data Protection Regulation (GDPR) and EU Regulation 2016/679.

### Measures

*Demographic characteristics*: We recorded participants’ age, ethnic background, level of education, number of children, marital/relationship status, number of years of victimization, and type of abuse (sexual abuse, physical abuse, psychological abuse).

*The Beck Depression Inventory* (BDI-II; Beck et al., 1996; Italian validation by Ghisi et al., 2006): Depressive symptoms were assessed using the BDI-II, a 21-item tool that covers the cognitive, affective, motivational and behavioral components of depression. Each item is rated on a four-point scale from 0 (never) to 3 (always). The total score (maximum 63 points) is the sum of the scores for the individual items. Based on the Italian validation study, a cut-off score of ≥12 was used to establish whether depression was present. Scores from 13 to 19 indicate mild depression; from 20 to 28, moderate depression; and from 29 to 63, severe depression. Cronbach’s α coefficient has ranged from 0.80 to 0.87 in normative or clinical samples (Beck et al., 1996). In this study, the α coefficient was 0.85 at Time 1 and 0.84 at Time 2.

*Los Angeles Symptom Checklist* (LASC; King et al., 1995). The LASC is a 43-item self-report instrument. It provides a measure of global distress due to trauma exposure, severity of overall PTSD symptomology, and severity of individual PTSD symptoms (re-experiencing, avoidance/numbing, and hyperarousal). Previous studies found high internal consistency with α coefficients ranging from 0.88 to 0.95 (King et al., 1995). In this study, the α coefficients were 0.91.

### Linguistic analysis

The narratives elicited during the three writing sessions were transcribed to analyze cognitive and emotional processing patterns linked to health improvements. The Linguistic Inquiry Word Count application (LIWC; Pennebaker et al., 2001, Italian vocabulary by Agosti and Rellini, 2007; Boyd et al., 2022) was used to assess language patterns and frequencies. The LIWC program calculates the frequency of words in a text. It recognizes approximately 2,000 words and codes them under a set of linguistic categories (such as pronouns, past, present and future tense, negative and positive emotion words, insight words, …). It calculates the total number of words in a text and computes the ratios of the different linguistic categories to overall corpus. In this study, we concentrated our analysis on words that reflected cognitive processing in terms of causal reasoning (e.g., *reason*, *because*, *thus*) and insight (e.g., *realize*, *see*, *understand*) and emotional processing in terms of positive emotion (e.g., *happy*, *joy*, *elation*) and negative emotion (e.g., *sad*, *mad*, *guilt*, *angry*).

### Statistical analyses

The descriptive analysis entailed computing participants’ baseline scores for PTSD and depression. There were no significant differences between the EW and NW groups at baseline.

Regarding the first research question, repeated-measure ANOVAs were run to test the effects of the EW intervention on the study outcomes as compared to the effects of the NW. All the ANOVA models included a within-subject factor (pre-scores and post-scores), a between-subjects factor (EW vs. NW), and their interaction. Statistically significant outcomes were further probed *via* plot analysis.

Regarding the second research question, focusing only on the EW group, delta values (∆) were computed for the differences between global pre-test and post-test scores for PTSD and depression, as well as for language-related variables. Delta values for cognitive and emotional processing were computed by subtracting the score for the first writing session from the score for the third writing session. Next, hierarchical multiple regression analysis was conducted as recommended by Baron and Kenny (1986). A mediational model would be deemed valid if four conditions were satisfied: the first and the second concern the effect of the predictor (1- depression baseline value for the first model, PTSD baseline value for the second model) on the dependent variables (∆ depression for the first model, ∆ PTSD for the second model) and (2) on the mediator variables (positive emotion and negative emotion processing and cognitive processing). The third (3) required a significant relationship between the dependent variables and the mediator variable after controlling for the specific effects of the predictor. When these conditions were satisfied, the first and the third regressions were compared to check the effect of the predictor on the dependent variable. The mediational model was accepted if (4) the effect of the predictor on the outcome was null (fully mediated) or lower (partially mediated) in the regression where the mediator was included.

All statistical analyses were conducted using SPSS 21.

## Results

### The EW effects

Statistically significant interaction effects were found for depression and PTSD symptoms. The plot analysis showed that: (1) depression symptoms decreased significatively in the EW group only (depression *F* = 0.551, *p* = 0.46; depression × writing condition *F* = 6.133, *p* = 0.03); (2) PTSD symptoms decreased in both groups but more in the EW condition (PTSD *F* = 10.745, *p* = 0.02; PTSD × writing condition: *F* = 10.145, *p* = 0.02; see Table 2).

**Table 2:** Repeated-measure ANOVAs.

	Sum of square	df	Mean square	*F*	*p*
Depression	3,178	1	3,178	0.551	0.463
Depression*depression effect	5,842	1	5,842	0.645	0.383
Depression*writing condition	34,873	1	34,873	6,133	0.038
PTSD	15,678	1	15,678	10,745	0.024
PTSD*PTSD effect	6,779	1	6,779	1,231	0.272
PTSD*writing condition	13,567	1	13,567	10,145	0.022

### Mediational models

Multiple regression analyses were performed in the EW group on the∆ values for depression, PTSD, positive emotion processing, negative emotion processing, and cognitive processing.

**Figure 1:**
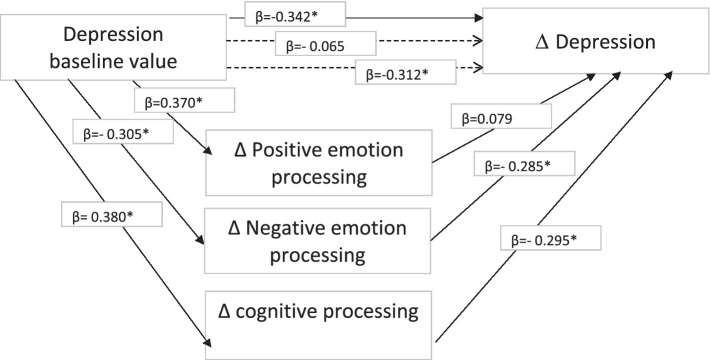
Mediational model for depression.

Concerning depression, the baseline score predicted reduced depression in the post-writing condition, with greater improvement displayed by those with more severe symptoms at time 1 (*β* = −0.342*; *R*_2_ = 0.117, *p* = 0.02). A higher level of positive emotion processing was predicted by higher baseline depression (*β* = 0.370*; *R*_2_ = 0.118, *p* = 0.02), but there was no mediational effect on reduced depression following writing. Higher baseline depression predicted less improvement in negative emotion processing (*β* = −0.305*; *R*_2_ = 0.123, *p* = 0.02), with negative emotion processing fully mediating changes in depression scores (*β* = −0.342* vs. *β* = −0.065). Higher baseline depression also predicted a lesser improvement in cognitive processing (*β* = 0.380*; *R*_2_ = 0.143, *p* = 0.02), and cognitive processing partially mediated the effect of baseline values on changes in scores at time 2 (*β* = −0.342* vs. *β* = −0.312*; see Figure 1).

**Figure 2:**
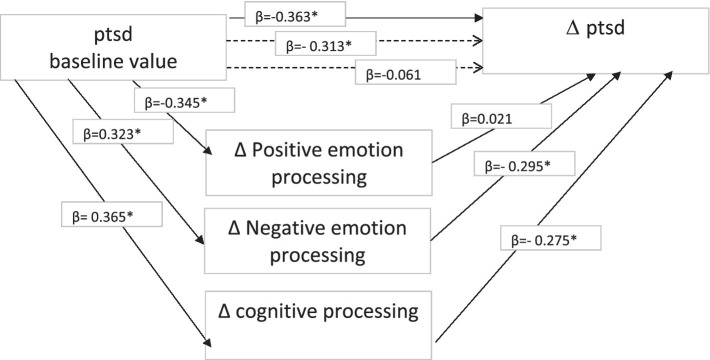
Mediational model for PTSD.

Concerning PTSD, a different pattern of functioning was identified. Reduced PTSD post- writing was predicted by baseline score, with the most significant improvement displayed by those with the most severe symptoms at time 1 (*β* = −0.363*; *R*_2_ = 0.147, *p* = 0.02). Higher baseline PTSD predicted greater improvement in positive emotion processing (*β* = −0.345*, *R*_2_ = 0.164, *p* = 0.02), but no mediational effect of positive emotion processing was found. Higher baseline PTSD predicted poor outcomes for negative emotion processing (*β* = 0.323*, *R*_2_ = 0.134, *p* = 0.02), and the effect of the baseline score was partially mediated by negative emotion processing (*β* = −0.363* vs. *β* = − 0.313*). Finally, higher PTSD scores pre-writing predicted poor cognitive processing (*β* = 0.365*, *R*_2_ = 0.184, *p* = 0.02), yet cognitive processing fully mediated the effects of baseline values on reducing PTSD after writing (*β* = −0.363* vs. *β* = −0.061; see Figure 2).

## Discussion

Our results confirm that expressive writing about traumatic experiences reduces depression and PTSD symptoms in female IPV victims. Consistently with previous studies (Pico-Alfonso et al., 2006; Ellsberg et al., 2008), women with histories of IPV reported significant depression and PTSD symptoms before engaging in EW, implying a need for intervention among this population. Our outcomes suggest that expressive narrative techniques improve health: women who wrote about their traumatic experiences while focusing on their emotion and thoughts enjoyed greater reductions in depressive and PTSD symptoms than their peers in the control group (Foà et al., 1995; Brown and Heimberg, 2001). Nevertheless, even the women in the control group who wrote about their traumatic experiences in exclusively factual terms displayed reduced PTSD following writing. Although intrusion has been reported to increase as a result of writing (Smyth et al., 2001), we hypothesize that being “forced” to think about traumatic experiences may reduce avoidance, which is associated with enhanced health. This happens among the EW group subjects because they are invited to write about their emotions and thoughts, but also in the neutral writing group because even writing factually fulfils an abreactive function. This explanation is in keeping with the Exposure Model and the use of exposure therapy for PTSD. Theoretically, being “forced” to confront negative experiences helps to overcome them, leading to health gains (for a review, see Foà et al., 2013). Regarding the specific mechanisms that may explain the beneficial effect of writing, our data confirmed a main effect of cognitive processing on improvements in depression and PTSD symptoms, in keeping with Cognitive Processing Theory. Change in the cognitive structure of narratives reflects an increase in cognitive processing. The repeated narration of trauma fosters reflection about its meaning, enhancing the subject’s sense of coherence and psychological well-being (Pennebaker and Seagal, 1999; Veronese et al., 2013; Castiglioni and Gaj, 2020). Cognitive processing helps traumatic memories to become ordinary memories, defusing their emotional intensity. This diminishes intrusive thoughts, enhances emotion regulation, and reduces arousal caused by stressful thoughts and memories (Davidson et al., 2002). The link between increased causal attributions and psychological improvements is also consistent with Ehlers and Clark’s (2000) finding that the ability to contextualize and process autobiographical memories promotes mental health in traumatized people. We may assume that producing narratives prompts reorganization of traumatic memories, enhancing the subject’s capacity to make sense of it. Meaning making mitigates the feelings of powerlessness and fragmentation experienced by IPV victims, who lost their sense of efficacy and control following the abuse.

We identified a different pathway for emotional processing. Specifically, stronger depression and PTSD before writing were associated with poor negative emotion processing. Nevertheless, success in decreasing negative emotionality protected subjects against the persistence of symptoms, especially depression, for which we found a full mediating effect (Sloan et al., 2007; Hoyt and Yeater, 2011). Differently to past research, which associated greater use of positive lexicon and moderate negative emotionality with health improvements (Pennebaker et al., 1997), we identified no mediating effect for positive emotion processing. This outcome may be interpreted in light of Holmes et al. (2007) who suggested that emotional processing in IPV survivors could be different than in other populations. More frequent expression of positive emotions may reflect decreased ability to actively cope with trauma, contributing to stress-related health problems. More frequent expression of positive emotion may thus mask defense mechanisms of avoidance and denial, as is typical in cases of interpersonal violence. Battered women often try to cope by playing down the seriousness of the abuse or underestimating its impact on themselves. They split negative affect from positive affect, exaggerating the latter (Bondura, 2016). In keeping with previous findings (e.g., Alpers et al., 2005), a strength of the present study is its use of automated text analysis with female victims of IPV, which should prompt broader research programs with clinical populations. Our work confirms the importance of viewing specific cognitive and emotional processes as predictive of health gains. It extends our understanding of how expressive writing interventions work and how to make them more efficacious. EW can be of value as a low-cost method that can also inform subsequent psychotherapy. Nevertheless, our study features methodological limitations. First, the small sample size reduces the generalizability of our results. Second, our sample comprised relatively highly educated participants (most had completed high school). Presumably, writing tasks are easier for the better educated, who may therefore receive greater benefit from it than less educated subjects. Also, PTSD and depression were assessed using self-report instruments, which may be undermined by respondent bias and inaccurate recall. Also, the sample was limited to women who had separated from their abusive partner and we do not know if the results would hold for women who remain in a violent relationship. Another limitation is the lack of a follow-up phase designed to test the long-term effects of writing. Finally, this study did not explore whether narrative benefits vary as a function of the specific subtypes of violent abuse suffered.

## Data availability statement

The raw data supporting the conclusions of this article will be made available by the authors, without undue reservation.

## Ethics statement

The studies involving human participants were reviewed and approved by E Campus University. The patients/participants provided their written informed consent to participate in this study.

## Author contributions

RP and MC together wrote the conclusions, specifically RP took care of the methodological part and MC took care of the initial part of the introduction. All authors contributed to the article and approved the submitted version.

## Conflict of interest

The authors declare that the research was conducted in the absence of any commercial or financial relationships that could be construed as a potential conflict of interest.

## Publisher’s note

All claims expressed in this article are solely those of the authors and do not necessarily represent those of their affiliated organizations, or those of the publisher, the editors and the reviewers. Any product that may be evaluated in this article, or claim that may be made by its manufacturer, is not guaranteed or endorsed by the publisher.
